# Miscellany

**Published:** 1910-09

**Authors:** 


### MISCELLANY

Medical Interne.—*Government Hospital for the Insane.*—The United States Civil Service Commission under date of August 13, announced an examination to be held October 5, 1910, at the usual places, to secure eligibles from which to make certification to fill a vacancy in the position of medical interne, Government Hospital for the Insane, Washington, D. C., at $600 per annum, with maintenance, and vacancies requiring similar qualifications as they may occur in that hospital, unless it shall be decided in the interest of the service to fill the vacancy by reinstatement, transfer, or promotion.

The positions are tenable for one year, and pay $50 a month and maintenance. At the end of six months, however, during which time a post-graduate course in mental and neurological diagnostic methods, and the like, is given, an examination is held, and promotions to the next grade, assistant physician, at $75 a month and maintenance, are made.

Applicants should at once apply either to the United States Civil Service Commission, Washington, D. C., or to the secretary of the board of examiners at any local place, for application Form 1312. No application will be accepted unless properly executed and filed with the commission at Washington. In applying for this examination the exact title as given at the head of this announcement should be used in the application.

As examination papers are shipped direct from the Commission to the places of examination, it is necessary that applications be received in ample time to arrange for the examination desired at the place indicated by the applicant. The Commission will therefore arrange to examine any applicant whose application is received in time to permit the shipment of the necessary papers.

